# Phase II study of the combination carboplatin *plus *celecoxib in heavily pre-treated recurrent ovarian cancer patients

**DOI:** 10.1186/1471-2407-11-214

**Published:** 2011-05-31

**Authors:** Francesco Legge, Amelia Paglia, Marco D'Asta, Gilda Fuoco, Giovanni Scambia, Gabriella Ferrandina

**Affiliations:** 1 Gynecologic Oncology Unit, Catholic University of Campobasso, Italy; 2 Gynecologic Oncology Unit, Catholic University of Rome, Italy

## Abstract

**Background:**

Cyclooxygenase-2 overexpression is associated with poor outcome and resistance to platinum-based chemotherapy in ovarian cancer. We evaluated the antitumor activity and safety of the combination carboplatin plus the COX-2 inhibitor celecoxib in recurrent heavily-treated OC patients.

**Methods:**

Patients were administered oral celecoxib (400 mg/day) in combination with intravenous carboplatin (AUC5, q28). A Simon's two-stage design was employed.

**Results:**

45 patients were enrolled: 23 (51.1%) presented platinum-resistance, and 27 (60%) had received at least 3 prior regimens for recurrence. The response rate was 28.9% with 3 complete and 10 partial responses (median duration of response = 6 months). Only one (0.4%) G4 non-febrile neutropenia was observed; G3 neutropenia, anemia, or thrombocytopenia, were observed in 2.5%, 1.7%, and 1.7% of the cycles, respectively. G3-4 vomiting was reported in only 1.7%, and 0.4% of the cycles were associated with G3 dyspepsia or diarrhea or constipation. Only one patient experienced G3 hypertension associated to G2 hypersensitivity reaction. No differences in baseline versus post-treatment Quality of Life scores were observed. Median progression free survival and overall survival were 5 and 13 months, respectively.

**Conclusions:**

Celecoxib combined with carboplatin showed promising activity and it is well tolerated in heavily-treated recurrent ovarian cancer patients.

**Trial registration number:**

NCT01124435 (ClinicalTrials.gov Identifier) and 935/03 (study ID numbers).

## Background

Most ovarian cancer (OC) patients experience recurrence of disease within 2 years from initial treatment, and typically are re-treated with platinum-based combinations, if considered platinum-sensitive (interval to recurrence/progression longer than 6 months) [1,2] or with non-platinum agents, such as liposomal doxorubicin, gemcitabine, topotecan, if considered platinum-resistant (interval to recurrence/progression less than 6 months from the completion of primary treatment) [3,4]. However, going on to receive multiple lines of chemotherapy, platinum re-treatment is very often attempted, also after the administration of several non-platinum drugs, and shows response rates ranging from 6 to 23% [5-7]. Given the palliative intent of any medical treatment of recurrent OC [8,9], the integration of non-cytotoxic drugs to standard chemotherapy has been proposed as a strategy to both increase response rates and/or decrease dose intensity and treatment related toxicity. In particular, novel strategies aimed at increasing platinum sensitivity should theoretically take advantage of targeting molecules not only involved in key steps of cancer biology such as proliferation/apoptosis balance, angiogenesis or immunosuppression, but also chemoresistance. In this context, cyclooxygenase-2 (COX-2), the key enzyme in prostaglandins (PGs) synthesis, seems to be a very suitable target, since it is involved in each of the above mentioned processes, it is overexpressed in tumors exhibiting pathological and clinical features of aggressiveness, and it is also associated with platinum-resistance and unfavorable prognosis in OC as well as in other human malignancies [10-14].

Indeed, selective COX-2 inhibitors have been shown *in vitro *and *in vivo *to exert a potent tumor growth inhibition not only in COX-2 positive tumors, but also indirectly in COX-2 negative tumors, through the growth inhibition of COX-2 expressing endothelial cells, and the positive modulation of immune functions [15,16]. Selective COX-2 inhibitors have been shown to be active as tumor chemopreventive agents in preclinical models as well as in humans [15,17,18], and to enhance the cytotoxicity exerted *in vitro *by different chemotherapeutics, including platinating agents [19,20].

The safety of celecoxib, which, amongst COX-2 inhibitors, exhibits the greatest potency for growth inhibition [21], has been extensively studied in patients with arthritis: at doses of 400 mg/day, celecoxib presents a toxicity profile similar to traditional non-steroidal inflammatory drugs, with the advantages of a reduced incidence of gastric ulcers and symptomatic gastrointestinal adverse events [22]. Although long-term use of COX-2 inhibitors has come recently under scrutiny due to the documentation of increased risk of serious cardiovascular events in patients treated with celecoxib at 400-800 mg/day, the hazard ratio for death from cardiovascular causes, has been reported to be 2.3 in the low dose group [23]. Even though it is unlikely that cardiovascular toxicity could affect the clinical outcome of poor prognosis recurrent OC patients, these data have been considered in the selection of the celecoxib's dose (400 mg/day) and in the eligibility criteria of the study.

Based on these evidences, we conducted a phase II clinical trial aimed at evaluating the antitumor activity and potential adverse effects of the combination celecoxib plus carboplatin in patients with recurrent, heavily pre-treated OC who had exhausted treatment options. The potential changes induced by the experimental combination on angiogenesis-related serum markers and quality of life measures were also evaluated.

## Methods

### Study population

This study was approved by the Institutional Ethical Committee of the Catholic University of Rome. The trial registration numbers for this phase II study are NCT01124435 (ClinicalTrials.gov Identifier) and 935/03 (study ID numbers). Eligible patients were required to have recurrent epithelial ovarian, fallopian tube, or peritoneal serous carcinomas with measurable disease as assessed by Response Evaluation Criteria in Solid Tumors (RECIST) criteria [24]. Patients were required to have received a platinum-containing regimen as primary treatment, at least one line of chemotherapy for recurrent disease. An interval time from the last platinum-based chemotherapy > 6 months, ≥18 years of age, Eastern Cooperative Oncology Group (ECOG) performance status of 0 to 2, adequate bone marrow (absolute granulocyte count ≥1,500/μl, hemoglobin >8.5 g/dl, and platelets count ≥100,000/≥30 ml/min), and hepatic (bilirubin ≤1.5 times upper limit of normal [ULN] and AST or ALT ≤3 times ULN) function were also required. Patients had to provide a written informed consent to the study protocol. Major exclusion criteria included: hypersensitivity to celecoxib, aspirin, other nonsteroidal anti-inflammatory drugs (NSAIDs), or sulfonamides; significant comorbidities (including any active coronary artery disease requiring management, symptomatic congestive heart failure, bleeding diathesis, uncontrolled severe hypertension, active gastrointestinal ulcer within 12 months, chronic inflammatory bowel diseases, deep venous or arterial thrombosis within 12 months, history of pulmonary embolism); concomitant use of possible interactive drugs (including chronic use of other NSAIDs); surgery, chemotherapy or radiotherapy within 1 month; actual or potential childbearing; breast-feeding; prior cancer treatment with a COX-2 inhibitor; any psychological, sociological or geographical condition potentially hampering compliance with the study protocol and follow-up schedule.

All eligible patients were included in the analysis of response, toxicity, quality of life (QoL), progression-free survival (PFS) and overall survival (OS) measures.

Primary and secondary platinum resistance have been defined as progression of disease within 6 months of completion of first line or salvage, respectively, platinum-based therapy. Platinum refractoriness is progression while on first line platinum-based therapy.

### Study design

This phase II prospective study was conducted at the Gynecologic Oncology Units of the Catholic University of Rome and Campobasso, Italy. The study was non-sponsored, investigators initiated. The primary objective was to determine the tumor response rate by RECIST criteria [24]. Secondary objectives included duration of response, progression-free survival (PFS), overall survival (OS), toxicity assessment, and QoL measures.

Patients were required to take celecoxib (200 mg tablets by mouth twice daily, day 1 to 28), in combination with intravenous carboplatin (area under the curve (AUC) 5 over 30 to 60 minutes, every 28 days). Patients who developed carboplatin hypersensitivity reaction (HSR) were allowed to follow a desensitization protocol, or alternatively to switch to cisplatin [25].

Erythropoietic stimulating agent and myeloid growth factors were not permitted for cycle 1 of study treatment, and their use was chosen by the treating physician, according to hospital policy.

### Toxicity and Efficacy

Before starting treatment, patients were evaluated by medical history, physical examination, cell blood count (CBC), chemistry panel, Ca125, and either computed tomography or magnetic resonance imaging scan. Toxicities were reported using the National Cancer Institute Common Terminology Criteria for Adverse Events version 3 [26]. Patients underwent weekly CBC and biweekly chemical panel during treatment. All laboratory tests were re-checked on day 1 of each cycle. Any patient receiving at least two cycles was assessable for tumor response, every three cycles, by RECIST criteria [24]. Clinical benefit was defined as a complete/partial response or a disease stabilization for at least 3 months. Toxicity was assessed at every cycle.

In addition, the criteria modified by Rustin [27] were used to define serological response: complete response was defined as the normalization of Ca125 serum levels to ≤35 U/ml confirmed by a second Ca125 measurement after 28 days, partial response was defined as a ≥50% decrease in Ca125 level after initiation of treatment confirmed 28 days apart, progression of disease was defined as a ≥50% increase in Ca125 level confirmed after 28 days, while stable disease was considered to be any response other than complete or partial response, or progression of disease.

Within 1 week before enrollment and every 3 cycles, QoL was assessed using the European Organization for Research and Treatment of Cancer Quality of Life Questionnaire C30 [28].

### Dose modifications and delay

To receive chemotherapy, patients needed to have an absolute granulocyte count of ≥1,500/μl, hemoglobin >8.5 g/dl, platelets count of ≥100,000/μl, and resolution of toxicities to ≤grade 1. No dose reduction was planned. Patients were removed from study if toxicities were not recovered by day 42 from the last cycle administration.

### Discontinuation of treatment

Treatment was discontinued when any of the following events occurred: radiographic or clinical evidence of cancer progression; deterioration of health or intolerable toxicity; patient refusal. Once patients stopped treatment, post-study treatment was dictated by the treating physician.

### Assessment of circulating levels of angiogenesis regulatory molecules

Plasma samples were collected in tubes containing heparin and serum was obtained using a serum separator tube (1500 RPM for 10 minutes at 4°C). All samples were aliquoted and stored at -80°C until assay. Serum levels of vascular endothelial growth factor (VEGF) and endostatin were measured by a solid phase chemiluminescent ELISA assay (R&D Systems, Abingdon, United Kingdom), according to the manufacturer's protocol [16].

### Statistical design and analysis

The primary endpoint was to determine the overall response (OR) rate. Secondary endpoints included the assessment of duration of response, PFS, OS, toxicity events and QoL scores. The OR rate, i.e. the combined rate of complete and partial responses, was reported as the proportion of the events among all patients, and was calculated via the intent-to-treat population. A Simon's two-stage accrual design was employed with an early stopping rule in the event that the treatment demonstrated insufficient activity. During the first stage of accrual, 13 patients were to be entered and evaluated. If at least 4 responses were observed among the first 13 patients, a second phase of accrual was to be initiated which would increase the accrual to 43 patients. The treatment would be considered active if at least 13 responses were observed among 43 patients. If the true probability of responding was only 20% (H0<13 responses), the study design provided a 95% chance of correctly classifying the treatment as inactive (alfa = 0.05). Conversely, if the true response rate was 40% (Ha≥13 responses), then the probability of correctly classifying the treatment as effective was 80% (beta = 0.8).

Duration of response was measured from the time that measurement criteria for response were met until the first date that progressive disease was documented. PFS and OS were measured from the date of start of study treatment to the date of evidence of progression or last seen, and the date of death from any cause or last seen, respectively. Toxicity events were summarized descriptively by frequency distribution.

Frequency counts and percentages were used to describe categorical variables, and median and range were used for continuous variables. Significance of differences between response rate in the different groups was calculated by the Fisher's exact test. Changes in QoL measures and VEGF and endostatin levels from baseline scores were compared using the Wilcoxon signed rank sum test. Survival probabilities were estimated according to the method of Kaplan and Meier [29]. Statistical analysis was carried out using SOLO (BMDP Statistical Software, Los Angeles, CA)

## Results

### Enrollment and Demographics

Between October 2003 and September 2007, 45 patients were enrolled.

Demographics and baseline characteristics of the study population are listed in Table 1. Most of the patients had serous histotype (76.7%), and poor grade of differentiation (80.6%). All patients were treated with platinum-based therapy including (82.2%) or not (17.8%) taxanes at time of initial diagnosis. Median progression-free interval from first line chemotherapy was 11 months (range, 2 to 66 months). Thirteen patients (28.9%) presented primary platinum-resistance, while 10 patients (22.2%) showed secondary platinum-resistance. The remaining 22 (48.9%) patients presented a "relative sensitivity" to platinum, since 10 cases achieved only a partial response to first line chemotherapy while 12 cases experienced disease progression within 6-12 months from the end of first line chemotherapy, according to previously defined criteria [3,5].

**Table 1:** Clinical/pathological characteristics of the study population (n = 45)

Characteristics	No (%)
**Histotype**	
Serous	33 (76.7)
Endometrioid	3 (7.0)
Clear cell	2 (4.6)
Undifferentiated	3 (7.0)
Other	4 (8.9%)

**Grade**	
G1-2	7 (19.4)
G3	29 (80.6)
n.a.	9

**First line Chemotherapy**	
Platinum-based	8 (17.8)
Platinum/taxane-based	37 (82.2)

**Platinum sensitivity**	
Primary refractoriness	4 (8.9)
Primary resistance	9 (20.0)
Secondary resistance	10 (22.2)
Relative sensitivity	22 (48.9)

**No. previous chemotherapy lines for recurrence treatment**	
1	6 (13.3)
2	12 (26.7)
3	19 (42.2)
≥4	8 (17.7)

**No. previous platinum-based re-challenges**	
None	25 (55.5)
1	17 (37.8)
2	3 (6.7)

**Age, years**	
Median (range)	59 (34-74)

**ECOG PS**	
0	20 (44.4)
1	23 (51.1)
2	2 (2.5)

Twenty-seven patients (60%) had received ≥ 3 prior chemotherapy regimens for recurrent disease. Median interval from the last platinum-based chemotherapy (primary or re-challenge) was 22 months (range, 8 to 48 months). At time of study enrollment, median age was 59 years (range, 34 to 74), and 44.4% of patients had an ECOG performance status of 0.

### Response Rates

Table 2 shows the results of treatment efficacy in the intent-to-treat population (n = 45), as well as in patients who received at least 2 cycles of the study treatment (n = 42). Indeed, three patients refuse to continue the treatment before two cycles were completed (see below).

**Table 2:** Response rates based on Intent-to-Treat population (n = 45) and in patients who have completed at least two cycles of the experimental combination (n = 42)

Characteristics	Intent-to-Treat patients No (%)	Patients completing at least two cycles of treatment No (%)
Patients	45	42

**Clinical response**		
Complete	3 (6.7)	3 (7.1)
Partial	10 (22.2)	10 (23.8)
Stable disease	13 (28.9)	12 (28.6)
Progression	19 (42.2)	17 (40.5)

**Duration of response, months** **Median (range)**	6 (3-13)	6 (3-13)

**Duration of stabilization, months** **Median (range)**	5 (3-10)	5 (3-10)

Serological Response*		
Complete	8 (19.0)	8 (20.5)
Partial	9 (21.4)	8 (20.5)
Stabilization	12 (28.6)	11 (28.2)
Progression	13 (31.0)	12 (30.8)

*Patients with 2 pre-treatment samples above the upper limit of normal (35 U/ml) Ca125 levels, and at least 2 additional samples after the start of treatment (n = 42).

In the intent-to-treat population, the overall response rate was 28.9% with 3 complete (6.7%), and 10 partial (22.2%) responses. Most responses (n = 11, 84.6%) were documented after 3 cycles of treatment; the median duration of response was 6 months (range, 3 to 13 months). Thirteen patients (28.9%) experienced stabilization of disease (median duration = 5 months, range 3 to 10 months), while 19 patients (42.2%) had progressive disease. Overall, 26 (57.8%) patients experienced a clinical benefit.

One (25%) response was observed among 4 platinum-refractory patients, 3 (15.8%) responses among 19 platinum-resistant patients and 9 (40.9%) responses among the 22 patients with relative sensitivity to platinum: although considering the limits of a small series, the response rate was not associated with platinum sensitivity (17.4% in resistant *versus *40.9% in relatively sensitive cases, p = 0.11). Moreover, the response rate, was not associated with the interval from the last platinum (data not shown).

No difference was noted in the response rates of patients who have completed at least two cycles of the experimental combination: indeed, objective response rate was 30.9% with a clinical benefit observed in 59.5% of the patients.

When considering the serological responses, we documented the return to the normal Ca125 levels, and the reduction ≥ 50% in Ca125 levels, in 8 (19.0%), and 9 (21.4%) patients, respectively; serological stabilization of disease was observed in 12 (28.6%) patients, totaling 29 (69.0%) patients not experiencing Ca125 increase during the study protocol (Table 2).

### Toxicities

Table 3 shows the study drugs administration details. In the whole study population a total of 238 cycles of platinum-based chemotherapy was administered, 196 of which included celecoxib; the median number of platinum plus celecoxib cycles per patient was 3 (range 1-10). Neither dose reductions, nor dose delays were recorded. Treatment withdrawal was registered for the following reasons: a) in 5 cases because of patient refusal due to G1 vertigo (n = 1), G1 motor neurotoxicity (n = 1), G3 carboplatin HSR and refusal of the de-sensitization protocol or re-challenge with cisplatin (n = 1), G3 diarrhea (n = 1), and G2 diarrhea associated with G2 rectal bleeding (n = 1): the last three patients experienced early toxicity during the first 5 weeks of treatment and refused further continuation of the experimental combination; b) in 4 cases because of toxicity including G3 hypertension associated to G2 HSR (n = 1), G2 skin desquamation (n = 1), G2 abdominal pain (n = 1), G3 dyspepsia (n = 1); c) in 28 patients (62.2%) due to progression of disease; d) in 8 patients after achieving response to treatment.

**Table 3:** Study drugs administration details

Total chemotherapy cycles administered	238
**Total chemotherapy + celecoxib cycles administered**	196

**Chemotherapy + celecoxib cycles per patient, median (range)**	3 (1-10)

**Reason for treatment withdrawal**	
Patient's refusal	5 (11.1)°
Toxicity	4 (8.9)°
Progression of disease	28 (62.2)
Patient's decision after confirmation of response	8 (17.8)*

°See Table 4 for details

* All patients submitted to ≥ 6 cycles of treatment.

Table 4 lists the toxicities observed. Only one case (0.4% of the cycles) of G4 hematological toxicity was observed, and no patient experienced febrile neutropenia. Grade 3 anemia, neutropenia, or thrombocytopenia, were observed in 1.7%, 2.5%, and 1.7% of the cycles, respectively. Only one patient was prescribed myeloid growth factor support at some point during therapy (7 cycles, 2.9% of the cycles); erytropoietin was prescribed in one patient (4 cycles, 1.7% of the cycles).

**Table 4:** Treatment-related toxicity graded by the Common Toxicity Criteria (version 3.0): overall assessment of adverse events per cycle (n=238)

Toxicity	Grade 1	Grade 2	Grade 3	Grade 4
**Hematological**				
**Anemia**	48 (20.2)	42 (17.6)	4 (1.7)	0
**Neutropenia**	32 (13.4)	38 (16.0)	6 (2.5)	1 (0.4)
**Thrombocytopenia**	10 (4.2)	14 (5.9)	4 (1.7)	0

**Gastrointestinal**				
**Nausea**	26 (10.9)	10 (4.2)	0	0
**Vomiting**	18 (7.6)	8 (3.4)	3 (1.3)	1 (0.4)*
**Dyspepsia**	7 (2.9)	0	1 (0.4)°	0
**Constipation**	8 (3.4)	6 (2.5)	1 (0.4)*	0
**Diarrhea**	5 (2.1)	7 (2.9)	1 (0.4)°	0
**Rectal bleeding**	0	1 (0.4)°	0	0
**Abdominal pain**	1 (0.4)	2 (0.8)°	0	0
**AST/ALT elevation**	4 (1.7)	0	0	0

**Neurological**				
**Hypoacusia**	1 (0.4)	1 (0.4)	0	0
**Sensitive Neurotoxicity**	2 (0.8)	0	0	0
**Motor Neurotoxicity**	2 (0.8) °	0	0	0

**Vertigo**	1 (0.4)°	0	0	0

**Fatigue**	13 (5.5)	6 (2.5)	2 (0.8)	0

**Venous thrombosis**	1 (0.4)	0	0	0

**Skin (erythema/desquamation)**	5 (2.1)	1 (0.4)°	0	0

**Hypertension**	0	1 (0.4)	1 (0.4) °	0

**Renal failure**	0	1 (0.4)	0	0

**Carboplatin HSR**	1 (0.4)	2 (0.8)	4 (1.7)°	0

* associated with progression of disease

°Reason for treatment withdrawal

As far as nonhematological toxicity is concerned, G3-G4 vomiting was reported in only 1.7% of cycles, while G3 dyspepsia, or diarrhea, or constipation were observed in 0.4% of cycles, respectively.

Six patients (13.3%) experienced carboplatin HSR during treatment (G2, n = 2; G3, n = 4): three patients had received prior platinum in the recurrent setting, whereas the remaining 3 had received platinum as part of the primary treatment. One patient refused further treatment, while the remaining 5 were switched to cisplatin. All patients experiencing G3 HSR required intravenous medication with diphenydramine and corticosteroids during the reaction.

### Quality of life measures

Overall, 32 patients (71.1%) completed the QoL questionnaire at baseline and at least one time after study treatment. For all scales/items, there was no statistically significant difference in baseline scores with respect to scores evaluated after three cycles of treatment: median global QoL score was 5 (range 2-7) versus 4.5 (range 1-7), at baseline and post-treatment, respectively (p value = 0.24). Moreover, the patterns of change in individual patients were not associated to clinical response (data not shown).

### Post-study treatments

Twenty-one (47%) patients were submitted to palliative care, in 16 (35%) oral/intravenous cyclophosphamide or etoposide was administered and 8 (18%) patients were treated with other cytotoxics such as weekly gemcitabine or taxanes.

### Survival

**Figure 1:**
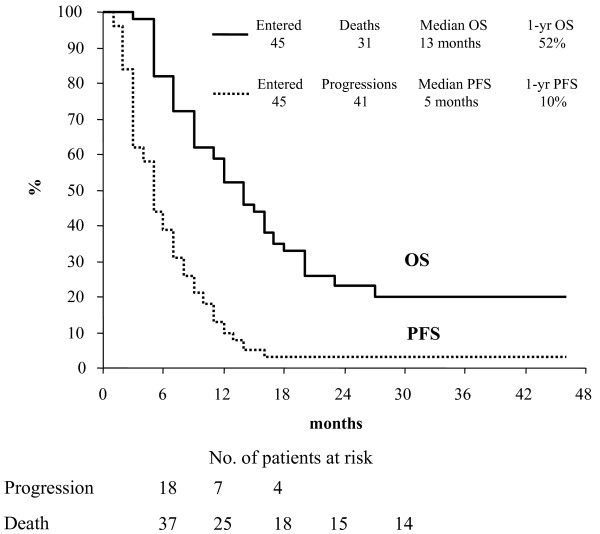
**Progression-free survival and Overall Survival in the study population (n = 45)**.

The median duration of follow-up was 12.0 months (range 3-46). PFS and OS curves for the study population are shown in Figure 1. Median PFS was 5 months with 39.2% of patients alive without disease progression after 6 months from the enrollment. Median OS was 13 months with 81.9% of patients alive after 6 months from the enrollment.

In the group of 26 patients experiencing a clinical benefit to the study treatment, a median PFS of 8 months and a median OS of 17 months was recorded.

### Assessment of circulating levels of angiogenesis regulatory molecules

Serum levels of VEGF and endostatin were evaluated in a preliminary series of 11 patients at baseline and after 1 month of carboplatin-celecoxib. Neither VEGF or endostatin levels resulted significantly changed in paired pre- and post-treatment samples: median VEGF serum level was 524 ng/ml (range 188-1,255) versus 420 ng/ml (range 208-1,215) at baseline and post-treatment, respectively (p value = 0.96); median endostatin serum level was 102 ng/ml (range 62-281) versus 90 ng/ml (range 7-184), at baseline and post-treatment, respectively (p value = 0.17). Moreover, the patterns of change in individual patients were not associated to clinical response (data not shown).

## Discussion

The combination celecoxib-carboplatin is active and well tolerated in patients with recurrent, heavily treated OC, with an overall response rate of 28.9% and a median PFS of 5 months.

These figures compare favourably with previously published results (response rate of 6-23%) obtained with platinum re-challenge in heavily treated recurrent OC patients [5-7]. We have to bear in mind that in our study the experimental treatment was administered in most patients as fourth line chemotherapy, and that half of cases were characterized by primary or secondary resistance to platinating agents.

The response rates were found not to be significantly associated with the grade of platinum-resistance, thus suggesting a potential influence of celecoxib in favourably modulating the susceptibility to platinating agents. One could argue that the activity documented in platinum-resistant and relatively sensitive subgroups is comparable to the amount of responses reported for platinum reinduction in these specific populations [5,7,30-32]. However, so far, it is difficult to directly compare our results with previously published studies due to the retrospective design of these series [5,7,30-32], the type of response assessment (i.e. serological *versus *radiological) [5,31], and the number of previously administered chemotherapy lines [30-32]: in order to definitively assess the potential role of celecoxib in enhancing platinum susceptibility in platinum-sensitive *versus *platinum-resistant patients a randomised trial and/or a larger and homogeneous series is required.

Previously reported *in vitro *studies showed the ability of selective COX-2 inhibitors to enhance cytotoxicity of platinating agents and this chemosensitization activity seems to be apoptosis-mediated and dependent from the levels of COX-2 expression [19]. Indeed, high intratumoral COX-2 expression has been associated with chemoresistance in different human cancer, including OC [10-14]. Moreover, we have recently shown that the association between COX-2 overexpression and chemoresistance is documented only for platinum-based therapy but not for platinum-taxane combination [12]. In this context, it is conceivable that the chemosensitizing activity of celecoxib could more clearly emerge in association with a single platinating agent. Indeed, it cannot be excluded that the discouraging results obtained in other studies investigating the chemosensitizing activity of celecoxib, may be also related to the use of platinum doublets including taxanes or antimetabolites [33-35] while, our study is the only one to our knowledge, investigating the combination of celecoxib with a single agent platinating compound.

Our data could appear even more interesting considering that the celecoxib doses we used (200 mg twice daily) are considered suboptimal compared to the doses (400 mg twice daily) previously approved for familial adenomatous polyposis prevention [18,36], and used in numerous clinical trials mostly under way [37]. However, whether lower doses of celecoxib are sufficient or not to maximally inhibit COX-2 activity is unknown: on the basis of our results, the dose of 400 mg/day can be considered clinically as adequate to the main purpose of chemosensitization. One can argue that we did not find in the sera of our patients any modulation of key angiogenesis-related factors, such as the pro-angiogenic VEGF and the anti-angiogenic endostatin, which have been previously associated with the antitumoral activity of celecoxib given at doses of 800 mg/day [16,38]. However, while higher doses are required to obtain antitumoral effects with celecoxib alone in terms of short-term modulation of molecular markers involved in tumor growth, apoptosis, immune function or angiogenesis [16], these could not be necessary for circumventing COX-2-mediated chemoresistance mechanisms in combinational study with chemotherapy.

As far as treatment safety is concerned, in our study an acceptable toxicity profile was documented. The chronic use of selective COX-2 inhibitors has been mainly associated with gastroduodenal perforations and intestinal bleeding [22]: however, in our study no severe gastrointestinal event occurred. Moreover, the addition of celecoxib did not seem to increase other toxicities classically associated with the administration of carboplatin, such as the haematological or neurological ones. As regards the main concerns recently emerged about the possible, dose-related, cardiovascular toxicity associated with chronic exposure to COX-2 inhibitors [23], notwithstanding enrolled patients were submitted to a median of 3 months of celecoxib-carboplatin, no serious cardiovascular events was reported in our study population: only one patients experienced a grade 1 venous thrombosis, recovering after two weeks of therapy. Another patient developed grade 3 hypertension associated with a grade 2 HSR, thus leading the patient to withdraw the protocol. In this context, since HSRs are reported in the literature in about 12-22% of patients submitted to platinum re-challenge, the rate and grade of severity of HSR observed in our combination study (i.e. 2.9% of cycles, 13.3% of the enrolled patients) seems acceptable [25].

This low rate of severe treatment-related complications, together with the acceptable toxicity profile, support the safety of the study combination, especially considering that our patients were heavily chemotherapy pre-treated, and a half of them were >60 years old. Moreover, the schedule of carboplatin 5 AUC every 4 weeks, administered in an outpatient setting, and the oral formulation of celecoxib allowed patients to stay at home, thus maintaining their family/social relationships, which are of utmost importance in patients with a short life-time expectancy. As a confirmation of the good tolerability of the study treatment, no QoL deterioration was observed in the overall population. Besides quality of life preservation, major goals in the treatment of heavily treated recurrent OC, include prolongation of survival: in the overall population, more than half of the patients survived at least 1 year from the enrolment in the study. Moreover, most of the patients (about 60%) experienced a long lasting clinical benefit, in terms of tumor response or stabilization, showing a median PFS of 8 months and a median OS of 17 months.

## Conclusions

Celecoxib combined with carboplatin in the platinum re-challenge of heavily-treated recurrent OC patients, showed promising activity and appeared well tolerated. This results could promote more tailored clinical trials, possibly randomised, aimed at drawing more definitive conclusions about the role of celecoxib in increasing platinum sensitivity in specific clinical settings. Further translational studies are necessary to better define the subgroups of patients taking advantages from the carboplatin-celecoxib combination. For instance, the urinary level of the major prostaglandin E2 metabolite, PGE-M (11α-hydroxy-9,15-dioxo-2,3,4,5-tetranor-prostane-1,20-dioic acid), has been reported as an effective biomarker at predicting and selecting patients that may respond to and benefit from COX-2 inhibition in combination with traditional therapies [38,39].

## Abbreviations

Cyclooxygenase-2: COX-2; Ovarian cancer: OC; Cyclooxygenase-2: COX-2; Prostaglandins: PGs; Response Evaluation Criteria in Solid Tumors: RECIST; Eastern Cooperative Oncology Group: ECOG; Nonsteroidal anti-inflammatory drugs: NSAIDs; Quality of life: QoL; Progression-free survival: PFS; Overall survival: OS; Hypersensitivity reaction: HSR; Cell blood count: CBC; Vascular endothelial growth factor: VEGF.

## Competing interests

The authors declare that they have no competing interests.

The authors have no any financial competing interests

The authors have no received reimbursements, fees, funding, or salary from an organization that may in any way gain or lose financially from the publication of this manuscript, either now or in the future.

The authors do not hold any stocks or shares in an organization that may in any way gain or lose financially from the publication of this manuscript, either now or in the future

The authors have no any non-financial competing interests (political, personal, religious, ideological, academic, intellectual, commercial or any other) to declare

## Authors' contributions

FL and GFe have made substantial contributions to the conception and design of the study, and to the analysis and interpretation of data. FL and AP have been involved in drafting the manuscript. MD and GFu have made substantial contributions to the acquisition and analysis of data. GS has been involved in revising the manuscript critically.

All authors have participated sufficiently in the work to take public responsibility for appropriate portions of the content.

All authors read and approved the final manuscript

## Pre-publication history

The pre-publication history for this paper can be accessed here:

http://www.biomedcentral.com/1471-2407/11/214/prepub
